# Grapevine-Associated Lipid Signalling Is Specifically Activated in an *Rpv3* Background in Response to an Aggressive *P. viticola* Pathovar

**DOI:** 10.3390/cells12030394

**Published:** 2023-01-21

**Authors:** Gonçalo Laureano, Catarina Santos, Catarina Gouveia, Ana Rita Matos, Andreia Figueiredo

**Affiliations:** BioISI–Biosystems & Integrative Sciences Institute, Faculdade de Ciências da Universidade de Lisboa, 1749-016 Lisbon, Portugal

**Keywords:** *Vitis vinifera*, downy mildew, fatty acids, phospholipase C, phospholipase D, C18:3

## Abstract

*Vitis vinifera* L. is highly susceptible to the biotrophic pathogen *Plasmopara viticola*. To control the downy mildew disease, several phytochemicals are applied every season. Recent European Union requirements to reduce the use of chemicals in viticulture have made it crucial to use alternative and more sustainable approaches to control this disease. Our previous studies pinpoint the role of fatty acids and lipid signalling in the establishment of an incompatible interaction between grapevine and *P. viticola*. To further understand the mechanisms behind lipid involvement in an effective defence response we have analysed the expression of several genes related to lipid metabolism in three grapevine genotypes: Chardonnay (susceptible); Regent (tolerant), harbouring an *Rpv3-1* resistance loci; and Sauvignac (resistant) that harbours a pyramid of *Rpv12* and *Rpv3-1* resistance loci. A highly aggressive *P. viticola* isolate was used (NW-10/16). Moreover, we have characterised the grapevine phospholipases C and D gene families and monitored fatty acid modulation during infection. Our results indicate that both susceptible and resistant grapevine hosts did not present wide fatty acid or gene expression modulation. The modulation of genes associated with lipid signalling and fatty acids seems to be specific to Regent, which raises the hypothesis of being specifically linked to the *Rpv3* loci. In Sauvignac, the *Rpv12* may be dominant concerning the defence response, and, thus, this genotype may present the activation of other pathways rather than lipid signalling.

## 1. Introduction

Viticulture is one of the most important agricultural practices in the world, representing a significant economic value worldwide. *Vitis vinifera* L. is the most well-known and disease-susceptible grapevine species. In viticulture, one of the major threats is caused by *Plasmopara viticola* (Berk. & M.A. Curtis) Berl. & De Toni, a biotrophic obligatory oomycete that causes downy mildew disease [1]. To prevent yield and quality losses, the current strategy relies on the intensive application of phytochemicals, representing more than 70% of the total amount of fungicides used in Europe [2]. This practice carries substantial negative effects, such as pollution, biodiversity loss and health problems. Another concerning issue is the development of fungicide-resistant strains, which are becoming more aggressive and capable of overcoming fungicide treatments [3].

The search for sustainable viticulture has led to the development of breeding programs. This strategy aims at improving agronomic traits such as resistance to pathogens. American and Asian wild *Vitis* species possess innate resistance against *P. viticola.* Several *P. viticola* resistance loci (*Rpv*) have been identified, and the introgression of these *Rpv* loci from wild *Vitis* species into *V. vinifera* is desired to produce crossing hybrids that are tolerant to this pathogen [4]. However, the process of obtaining hybrids is extremely time-consuming and laborious. In addition, the emergence of new strains of *P. viticola* has been challenging the resistance of these hybrids [5]. Therefore, a deeper knowledge of grapevine defence mechanisms is essential, not only to accelerate breeding processes but also to define more sustainable disease control measures.

In the past twenty years, grapevine responses to *P. viticola* have been thoroughly characterised at the transcriptomic, metabolomic and proteomic levels [6,7,8,9,10,11]. Lipid involvement in grapevine tolerance to *P. viticola* has only recently been pointed out [12,13,14,15]. Lipids are multifaceted molecules carrying out not only structural and energy storage functions but also playing important signalling roles in plant defence (reviewed in [16]). Upon pathogen attack, cellular membranes undergo changes in their lipid and fatty acid (FA) composition which affect membrane properties such as permeability and fluidity [17]. In parallel membrane lipids, besides their structural roles, are also the substrates of lipid-metabolising enzymes, providing important signalling molecules such as free FA, phosphatidic acid (PA), inositol-3-phosphate (IP3) and oxylipins (reviewed in [16]). Moreover, lipid-metabolising enzymes, particularly phospholipases, catalyse the hydrolysis of acyl esters and phosphate esters in phospholipids. In plants, the three major families of phospholipases, A (PLA), C (PLC) and D (PLD), are classified according to the position in which they catalyse the hydrolysis of phospholipids. In contrast to PLC and PLD, which act on the polar head of phospholipids, PLA catalyses the hydrolysis of glycerolipids into lysolipids and free fatty acids, either at the sn-1 (PLA1) and/or sn-2 position (PLA2) of glycerolipids, whereas no-specific PLA can remove both FA from glyco- and phospholipids [18]

Lipid involvement in grapevine resistance against *P. viticola* was only recently unveiled. We have previously shown that during the first hours of interaction between *P. viticola* and the *Rpv3.1*-tolerant host, Regent, lipid modulation occurs followed by the activation of several biosynthetic and lipid signalling pathways. The main alterations take place at the chloroplast, where a high accumulation of α-linolenic acid (C18:3) is observed, increasing membrane fluidity [12,13]. This unsaturated FA is the bioprecursor of jasmonic acid (JA), a phytohormone involved in plant defence, which has a prominent role in the establishment of the incompatible interaction with *P. viticola* [14,15]. We have also shown that several desaturases, which introduce double bonds in fatty acids, and phospholipases A, which hydrolyse membrane lipids generating free FA, such as C18:3, are specifically upregulated during the interaction, highlighting their roles in grapevine defence [12,13]. The roles of PLC and PLD in plant defence have been described for other pathosystems but not yet in grapevine [19,20].

To increase our understanding of lipid signalling events in grapevine defence against *P. viticola* and to evaluate if these mechanisms are commonly activated in hosts with different genetic backgrounds, in this work, we have analysed three *V. vinifera* cultivars (‘Chardonnay,’ a susceptible genotype, ‘Regent’, a tolerant genotype harbouring *Rpv3-1* loci and ‘Sauvignac’, a resistant genotype harbouring a pyramid of *Rpv3-1* and *Rpv12* loci). As several reports have highlighted the appearance of more aggressive *P. viticola* pathovars, we have used the *P. viticola* isolate NW-10/16, which is able to partially overcome the *Rpv3* loci. We have conducted an expression analysis of several genes coding for enzymes involved in lipid metabolism and signalling and characterised the fatty acid modulation in grapevine during the first hours of inoculation with *P. viticola.*

Our work reveals, for the first time, the interplay between lipid signalling and resistance against downy mildew associated with an *Rpv* background.

## 2. Materials and Methods

### 2.1. Grapevine PLC and PLD Gene Family Characterization

#### 2.1.1. Grapevine PLC and PLD Gene Identification

The grapevine PLC and PLD genes were identified through homology searches with *Arabidopsis thaliana*, *Glycine max* and *Oriza sativa* PLC and PLD proteins. Both PLC and PLD sequences from these plants were obtained in the Tair [21] (https://www.arabidopsis.org/), accessed on 1 November 2022, Phytozome [22] (https://phytozome-next.jgi.doe.gov/, accessed on 1 November 2022) and NCBI databases (https://www.ncbi.nlm.nih.gov/) (accessed on November 2022), respectively. Grapevine PLC and PLD gene positions on chromosomes were determined using the genome annotations of Grape from the NCBI database (https://www.ncbi.nlm.nih.gov/, accessed on 1 November 2022), and the gene intron/exon structure information was also collected from the NCBI database. The molecular weight (Mw) and isoelectric point (pI) were predicted using the Compute pI/Mw tool from ExPASy [23] (https://web.expasy.org/compute_pi/, accessed on 1 November 2022).

#### 2.1.2. Phylogenetic and Domain Analysis

The phylogenetic trees were viewed on FIGTree (http://tree.bio.ed.ac.uk/software/Figtree/, accessed on 1 November 2022) and edited on Inkscape (http://www.inkscape.org/, accessed on 1 November 2022). The HMMER web server (https://www.ebi.ac.uk/Tools/hmmer/search/hmmscan, accessed on 1 November 2022) was used to determine the structures of grapevine PLC and PLD proteins by analysing the domains detected. To align the PLC and PLD protein sequences from *V. vinifera*, *A. thaliana*, *O. sativa* and *G. max*, the MAFFT software with the E-INS-I option [24] (http://mafft.cbrc.jp/alignment/software, accessed on 1 November 2022) was used. Maximum-likelihood phylogenetic analysis was performed with RAxML-HPC v8, according to previously adjusted conditions [25].

#### 2.1.3. Cis-Element Analysis

Predicted cis-acting elements were identified using the PlantCARE database [26] (http://bioinformatics.psb.ugent.be/webtools/plantcare/html/, accessed on 1 November 2022) in the genomic sequences, 2000 bp upstream of each gene coding region, searched on the NCBI database (https://www.ncbi.nlm.nih.gov/, accessed on 1 November 2022).

#### 2.1.4. Putative Functions and Subcellular Targeting Prediction

PLC and PLD putative functions, represented by GO terms, were predicted using the Blast2GO version 6.0 software tool [27] (https://www.blast2go.com/, accessed on 1 November 2022) and the newest tool from Google, ProteInfer [28] (https://google-research.github.io/proteinfer/, accessed on 1 November 2022). The subcellular location was predicted using TargetP–2.0 [29] (https://services.healthtech.dtu.dk/service.php?TargetP-2.0, accessed on 1 November 2022), Predotar [30] (https://urgi.versailles.inra.fr/predotar/, accessed on 1 November 2022), PredSL [31] (http://aias.biol.uoa.gr/PredSL/, accessed on 1 November 2022) and Localizer [32] (http://localizer.csiro.au/, accessed on 1 November 2022).

Gene localisation on grapevine chromosomes was also compared to the genetic linkage map representing *P. viticola* resistance loci in grapevine (*Rpv* loci; https://www.vivc.de, accessed on 1 November 2022) to access the location of the grapevine PLC and PLD genes within those loci.

### 2.2. Plant Material, RNA Extraction and cDNA Synthesis

Three *Vitis vinifera* cultivars with different resistant degrees to *P. viticola* were selected for this work, the susceptible cultivar Chardonnay (ViVC number 2455), the tolerant cultivar Regent (ViVC number 572) that harbours *Rpv3* loci and the resistant cultivar Sauvignac (ViVC number 22322) that harbours a pyramid of both *Rpv3* and *Rpv12* loci. These genotypes were grown under greenhouse conditions (22 °C/ day and 18 °C/ night) [13]. The potted grapevine plants were regenerated from wood cuttings obtained from the State Education and Research Centre of Viticulture, Horticulture and Rural Development, Neustadt/Weinstr., Germany, as described by Merz et al. [33].

Ten plants from each cultivar were used, and leaf discs were taken from three leaves per plant (third to fifth from the shoot apex). The leaf discs were randomly distributed in Petri dishes and inoculated with the *Plasmopara viticola* isolate NW-10/16 as described in [34].

This *P. viticola* isolate was recovered from field-grown Regent plants in Germany. This isolate can surpass *Rpv3*-associated resistance but not an *Rpv3*/*Rpv12* pyramid-associated resistance [34]. Inoculated and mock-inoculated leaf discs were harvested at 0, 4, 6, 12 and 24 h post-inoculation (hpi), immediately frozen in liquid nitrogen and stored at −80 ℃. Three biological replicates were collected per condition.

Total RNA was isolated with the Eurx Universal RNA extraction kit (Eurx ^®®^, Gdańsk, Poland) following the manufacturer’s instructions. The RNA purity (A260/A280 nm) and quality were measured using a Nanodrop 1000 spectrophotometer (Thermo Fisher Scientific Inc., Wilmington, DE, USA). To ensure a clean RNA sample, a second DNAse digestion with the Baseline-ZERO DNase (Biosearch Technologies, Lucigen, Middleton, WI, USA) was performed in all samples using 3 µg total RNA, 1 U of DNAse I and 1X Baseline-ZERO reaction buffer in a 100 µL reaction according to the manufacturer’s indications. The RNA was then quantified with a Qubit fluorometer using the Qubit RNA BR Assay Kit (Life Technologies Corporation, ThermoFisher Scientific). For cDNA synthesis, 500 ng of total RNA was reverse transcribed using the iScript™ cDNA Synthesis Kit (Bio-Rad Laboratories, Inc.).

### 2.3. Quantitative Real-Time PCR

The qPCR experiments were performed in a StepOne™ Real-Time PCR system (Applied Biosystems, Sourceforge, USA) using the Maxima™ SYBR Green qPCR Master Mix (2×) kit (Fermentas, Ontario, ON, Canada), according to the manufacturer’s instructions. For all genes, the PCR cycling parameters were thermal cycling started with a 95 °C denaturation step for 10 min followed by 40 cycles of denaturation at 95 °C for 15 s and annealing/ elongation at gene-specific temperatures (Table S1) for 30 s. Non-template controls were added. To confirm single-product amplification and the existence of non-specific PCR products, a dissociation curve analysis was performed (Figure S1). Gene expression (fold change) was calculated as described in Hellemans et al. [35]. For expression data normalisation, Elongation Factor 1-alpha (*EF1α*) and Ubiquitin-conjugating enzyme (*UBQ*) coding genes were used, as described previously [36,37].

### 2.4. Lipids

Fatty acids methyl esters (FAMEs) were prepared by the direct trans-methylation of the leaf disc samples (4, 6 and 24 hpi) using a solution of methanol:sulfuric acid (97.5:2.5, *v*/*v*) and incubating for 1 h at 70 °C. FAMEs were recovered in the organic phase, by adding petroleum ether:ultrapure water (3:2, *v*/*v*). The quantitative analysis of FAMEs was performed using Gas Chromatography (PerkinElmer Clarus^®®^ 590 GC), according to previously optimised conditions and chromatograms analysed using PerkinElmer Clarus 590 GC, version 6.3.3.0691 [38]. Heptadecanoic acid (C17:0) was used as an internal standard [25,39].

### 2.5. Statistical Analysis

Statistical analyses of all data were carried out by the Mann–Whitney U test using IBM^®®^ SPSS^®®^ Statistics software (version 23.0; SPSS Inc., Chicago, IL, USA). The qPCR statistical analysis was performed by Tukey’s multiple comparisons tests. Differences in the expression of a specific gene, within genotypes at each time point, were marked with a, b or c, according to their differences. The statistical analysis regarding fatty acid quantification was based on non-parametric tests due to the lack of data normality and homogeneity of variances. Results with a *p*-value < 0.05 were considered statistically significant.

## 3. Results

### 3.1. Grapevine PLC and PLD Gene Families Identification

#### 3.1.1. Grapevine PLC 

In plants, two major PLC classes were described, according to substrate specificity: the phosphoinositide-specific PLC (PI-PLC) and the phosphatidylcholine-cleaving PLC (PC-PLC), also known as nonspecific PLC (NPC), as that may use either phosphatidylcholine (PC), phosphatidylethanolamine (PE) or phosphatidylserine (PS) as substrates [19,40,41,42].

A total of eleven grapevine putative PLC genes coding for thirteen putative proteins, of which, eight proteins are PI-PLCs and five are NPCs (Table S2), were found. Four PI-PLCs are in chromosome 4, two in chromosome 8 and the other two in chromosome 13, whereas NPC members were distributed evenly in chromosomes 6, 15 and 17 (Table S2). Regarding the number of exons detected in the PLC family, the number varied between ten and three, being distributed differently in PI-PLC and NPC. In PI-PLC members, the isoforms *VviPI-PLC4.1* and *VviPI-PLC4.2* presented ten exons, *VviPI-PLC5* showed three, and the remaining PI-PLC genes showed nine exons. In the NPC group, five exons were observed in the isoforms *VviNPC3.1* and *VviNPC3.2*, three exons in the *VviNPC2* and *VviNPC4*, and four exons were detected in *VviNPC1* (Table S2).

*VviPI-PLC1* is in the *Rpv20* loci at chromosome 6, *VviPI-PLC7* is located in the *Rpv17* at chromosome 8, and *VviNPC3* is located in the *Rpv23* and *Rpv26* at chromosome 15.

#### 3.1.2. Grapevine PLD 

Phospholipases D catalyse the hydrolysis of structural phospholipids, such as PC and PE, to produce PA and the respective headgroup [43]. These PLs have been identified as an essential component of many cellular and subcellular processes, such as signal transduction and membrane degradation [44], being also involved in lipid metabolism and cellular regulation, including responses to both biotic and abiotic stresses [45]. This family is divided into three different classes, C2-PLD, PXPH-PLD and SP-PLD, depending on the N-terminal domain present in the protein as well as other properties. The members of the C2-PLD group have a Ca^2+^-dependent phospholipid-binding C2 domain (requiring Ca^2+^ for activity) [46] and hydrolyse various common membrane phospholipids such as PC, PE, PS and phosphatidylglycerol (PG) [47]. PXPH-PLD are Ca^2+^-independent enzymes presenting Phox homology (PX) and Pleckstrin homology (PH) domains, which are protein regulatory modules involved in cell signalling [44,48]. The last group, SP-PLD, also includes Ca^2+^-independent enzymes which have a signal peptide at the N-terminal [49].

In 2010, Qi Liu and co-workers characterised the PLD family in grapevine, for the first time identifying 11 PLD genes encoding for 11 proteins [50]. However, in 2011 a reannotation of the grapevine genomic information was performed. In order to update the previous information, a new characterisation of this gene family was conducted. A total of 11 putative genes coding for 15 putative proteins (13 C2-PLDs, 1 PX-PLD and 1 SP-PLD) were identified (Table S3). PLD genes are randomly and unevenly distributed on 8 out of 19 *V. vinifera* chromosomes. The C2-PLD genes were found on chromosomes 2, 4, 9, 11, 12, 15 and 18, whereas *VviPLDζ* and *VviPLDφ* were predicted to be located on chromosomes 5 and 4, respectively (Table S3). The exon–intron structure analysis of the PLD genes demonstrated that the highest exon number is 20, belonging to PXPH-PLD, followed by the C2-PLDs, where the number of exons ranged from 3 to 12, and finally, the SP-PLD, presenting 7 exons (Table S3).

*VviPLDα3* is in the *Rpv4* at chromosome 4, *VviPLDβ* is located in the *Rpv26* at chromosome 15 and *VviPLDζ* is located at *Rpv11* in chromosome 5.

#### 3.1.3. Phylogenetic Analysis and Putative Functions

**Figure 1:**
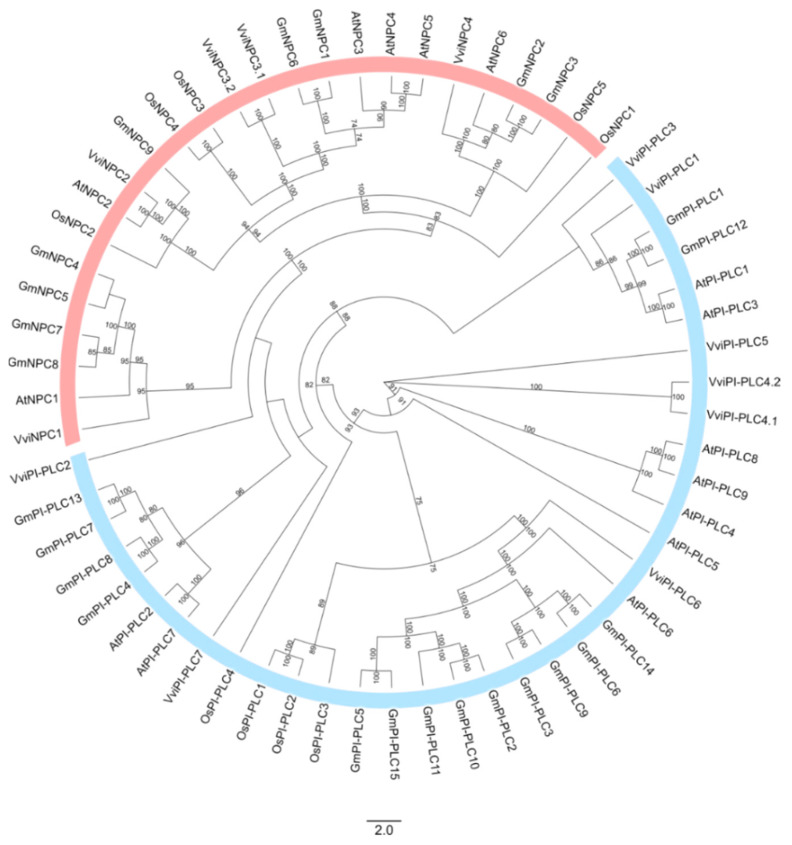
Maximum likelihood phylogenetic tree based on 61 Phospholipases C proteins from *Arabidopsis thaliana* (At), *Oriza sativa* (Os), *Glycine max* (Gm) and *Vitis vinifera* (Vvi). Different groups are represented by different colours: NPC in red and PI-PLC in blue. The numbers above the branches show bootstrap values. The scale bar represents the number of estimated changes per branch length.

**Figure 2:**
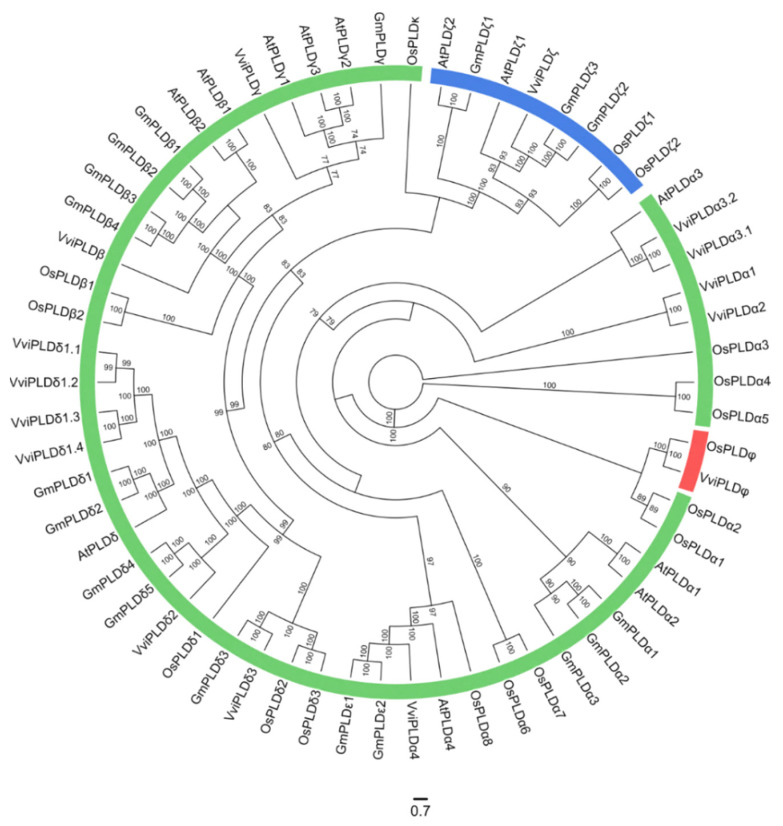
Maximum likelihood phylogenetic tree based on 62 Phospholipases D proteins from *Arabidopsis thaliana* (At), *Oriza sativa* (Os), *Glycine max* (Gm) and *Vitis vinifera* (Vvi). Different groups are represented by different colours: C2-PLD in green, PXPH-PLD in blue and SP-PLD in red. The numbers above the branches show bootstrap values. The scale bar represents the number of estimated changes per branch length.

Two independent phylogenetic analyses of both PLC and PLD were performed using protein sequences from *Vitis vinifera*, *Arabidopsis thaliana*, *Glycine max,* and *Oriza sativa*. According to the sequence identity with Arabidopsis, soybean and rice, the structure of the grapevine PLC (Figure 1) and PLD (Figure 2) families was predicted as well as the nomenclature proposal for each family member, considering the grapevine gene nomenclature method proposed by Grimplet and co-workers [51].

The grapevine PLCs were distributed in two major groups, the PI-PLC and the NPC. PI-PLC harbours eight members, (VviPI-PLC1, VviPI-PLC2, VviPI-PLC3, VviPI-PLC4.1, VviPI-PLC4.2, VviPI-PLC5, VviPI-PLC6 and VviPI-PLC7), whereas the NPC is composed of five members (VviNPC1, VviNPC2, VviNPC3.1, VviNPC3.2 and VviNPC4) (Figure 1).

The grapevine PLD family was distributed into three subfamilies: C2-PLD, PXPH-PLD and SP-PLD (Figure 2). The C2-PLD is the most represented subfamily, with 13 members (VviPLDα1, VviPLDα2, VviPLDα3.1, VviPLDα3.2, VviPLDα4, VviPLDβ, VviPLDγ, VviPLDδ1.1, VviPLDδ1.2, VviPLDδ1.3 VviPLDδ1.4, VviPLDδ2 and VviPLDδ3), whereas the PXPH-PLD and SP-PLD subfamilies are the least represented, having only one member each (VviPLDζ and VviPLDφ, respectively) (Figure 2).

#### 3.1.4. Identification of Cis-Elements of Grapevine PLC and PLD Genes

The conservative core promoter/enhancer elements CAAT-box and TATA-box were identified in all PLC and PLD members (Table S4). Cis-elements related to the abiotic factors’ response were found in both families: water response (MYB and AT-rich element) in 10 PLC and in all PLD genes, drought stress (MYC, as-1, MBS and DRE core) in all PLC and PLD members, low-temperature response (LTR) found in 5 PLC and 7 PLD genes, heat, osmotic stress, low pH and nutrient starvation stress response (STRE and TCA) in 9 PLCs and 8 PLDs, circadian response (circadian, ERE and ABRE) in all PLCs and in 12 PLDs and, finally, cis-elements related to light response (GT1-motif, TCT-motif, GATA-motif, MRE, AE-box, I-box, TCCC-motif, ATCT-motif, AT1-motif, GA-motif, Chs-CMA1a, 3-AF1 binding site, ACE, Gap-box, Sp1, Box 4, Box II, AAAC-motif, CAG-motif, ACA-motif, G-box, ATC-motif and Chs-Unit 1 m1) were identified in all genes belonging to both groups. AP-1, a cis-element involved in cadmium responsiveness, was only found in *VviNPC1*.

Several cis-acting elements associated with phytohormone response were also found in both PLC and PLD family members: the response to ethylene (ERE) in 9 PLCs and 11 PLDs, abscisic acid (ABRE, ABRE3a, ABRE4 and AT~ABRE) in 6 PLCs and 11 PLDs, gibberellic acid (P-box, TATC-box, GARE-motif and CARE) in 5 PLCs and 10 PLDs, jasmonic acid (CGTCA-motif and TGACG-motif) in 4 PLCs and 7 PLDs, salicylic acid (TCA-element) in 4 PLCs and 7 PLDs and to auxin (TGA-element, TGA-box and AuxRR-core) in 3 PLCs and 2 PLDs.

Regarding the response to wounding and pathogens, the cis-elements W-box, WUN-motif, WRE3, box S and TC-rich repeats were identified in all PLC and PLD genes, except in VviPLDφ, which is the only member belonging to SP-PLD family. Both ARE and GC-motif are related to anaerobic response (anoxic inducibility) and were also found in all members, except in *VviNPC1*. Some cis-elements involved in the cell cycle and cell proliferation response (Myb-binding site, CCGTCC-motif, CCGTCC-box, MSA-like, re2f-1, Box III, F-box, MBSI, CCAAT-box and dOCT) were found in all PLCs and PLDs, except for *VviPLDα4*.

MYB-related binding elements, such as MBS, MRE and MBSI117 were identified in eight members of both PLC and PLD families. MYB-related tissue-specific/preferentially expressed elements, associated with plant development and growth, were observed in all PLCs and in 10 PLDs: RY-element, involved in seed regulation; GCN4-motif, which regulates endosperm expression118; HD-Zip 1, generally involved in responses related to abiotic stress, abscisic acid, blue light, de-etiolation and embryogenesis123; O2-site, related to zein metabolism regulation; CAT-box, involved in plant growth and meristem expression AAGAA-motif and CTAG-motif, both with an unknown specific function.

#### 3.1.5. Protein structure and Domain Analysis

The protein size of the grapevine PLC ranged from 484 to 666 amino acids, with an average length of approximately 583 amino acids in the PI-PLC subfamily and 519 in the NPC. The predicted molecular weight (Mw) of the grapevine PI-PLC varies between 54.94 and 75.86 kDa and in NPCs from 57.43 to 60.04 kDa. The isoelectric point (pI) of the grapevine PLC proteins varied from 5 to 8.75, with an average of 6.38 (Table S2). Concerning the protein size of the grapevine PLD family, the highest value was found in PXPH-PLD members, with 1113 aa, being the lowest size detected in the SP-PLD, with 517 aa, whereas the C2-PLD presented a protein size comprised between 765 to 1087 aa. The predicted Mw varies between 57.99 kDa and 126.92 kDa, in which the highest Mw is from *VviPLDζ* and the lowest is from *VviPLDφ*. The grapevine PLD pI oscillated from 6.34 and 8.43, with an average of 6.7 (Table S3).

**Figure 3:**
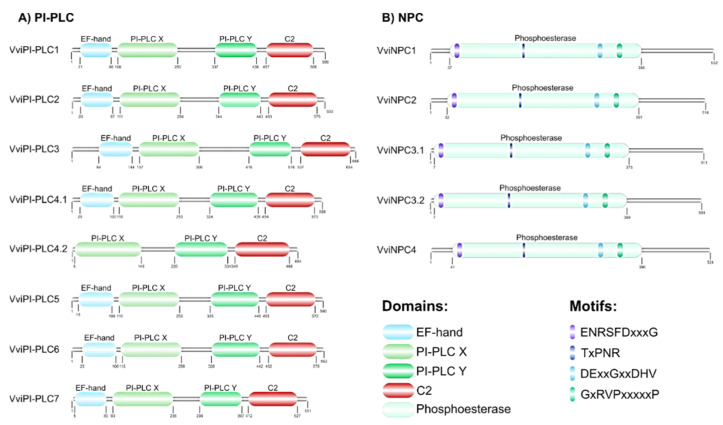
Peptide domain structures and conserved motifs of grapevine PLC members. Schematic diagram representing: (**A**) PI-PLC and (**B**) NPC groups. Different motifs and domains are indicated by different colours.

In grapevine, the PLC protein sequences exhibit distinct domains and motifs that are highly conserved in each subfamily. The PI-PLC presented four characteristic domains, EF-hand, PI-PLC X, PI-PLC Y and the C2 domain, except for VviPI-PLC4.2 which does not possess the EF-hand domain. Regarding the motifs, none were found in PI-PLC. In the NPC members, only one domain was found, the Phosphoesterase domain, and four motifs were predicted, ENRSFD(x)3G, TxPNR, DE(x)2G(x)2DHV and GxRVP(x)5P (Figure 3).

**Figure 4:**
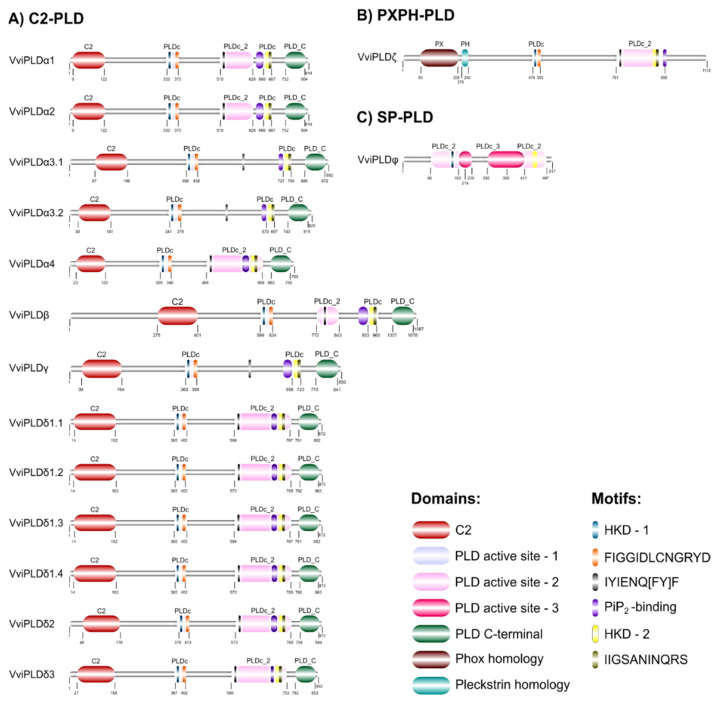
Peptide domain structures and conserved motifs of grapevine PLD members. Schematic diagram representing: (**A**) C2-PLD, (**B**) PXPH-PLD and (**C**) SP-PLD families. Different motifs and domains are indicated by different colours.

Contrary to the PLD subfamilies that have no domain or motif in common, the PLC subfamilies share some features. In grapevine, the active site 2 domain was observed in all PLD proteins, whereas the active site 1 was detected only in two subfamilies, C2-PLD and PXPH-PLD, and the active site 3 was only found in the SP-PLD. These active sites play an important role in PLD catalysis [52]. In C2-PLD, two specific domains, C2 and C-terminal, were only observed in this family. The VviPLDζ, the only member of the grapevine PXPH-PLD family, also presented two characteristic domains at the N-terminal, PX and PH domains (Figure 4).

The analyses also showed that all grapevine PLDs possessed two characteristic and structurally conserved HKD motifs: HKD-1, with 8 amino acids (HxK(x)4D) and HKD-2, with 18 amino acids (HxK(x)4D(x)6GSxN). With the exception of VviPLDφ, all grapevine PLD members presented four different motifs, FIGGIDLCNGRYD, IYIENQ[FY]F, IIGSANINQRS133 and PiP2-binding (Figure 4).

#### 3.1.6. Subcellular Targeting Prediction

PI-PLC proteins were predicted to be in the mitochondria (VviPI-PLC1, VviPI-PLC2, VviPI-PLC4 and VviPI-PLC6) and in the cytoplasmic membrane (VviPI-PLC3, VviPI-PLC4.2, VviPI-PLC5 and VviPI-PLC7) (Table S5). Regarding the PLD, only two proteins had their cellular locations predicted, the VviPLDα4 being predicted to be in the mitochondria, and the VviPLDφ, possessing a signal peptide, was predicted to be secreted. The remaining PLD proteins did not present a conclusive subcellular localisation. Blast2GO was also used to confirm the subcellular location and to confirm that the predicted functions were associated with lipid processing (Table S6).

### 3.2. Expression Analysis

A total of 12 genes were selected for expression analysis using the following criteria: previously described as being associated with defence against pathogens; *cis*-elements associated with JA response, wounding and pathogen/defence response and on the chromosomal location (within *Rpv* loci).

Five selected genes belong to the PLC superfamily (phosphoinositide-specific: *VviPI-PLC1*, *VviPI-PLC2*, and *VviPI-PLC7*, two nonspecific: *VviNPC3* and *VviNPC4*); three belong to the PLD superfamily (two C2-PLD: *VviPLDα3* and *VviPLDβ*, and *VviPLDζ*) and four genes already described by Laureano and colleagues [12,13] (the membrane-bound fatty acid desaturases *VviFAD8* and *VviFAD2-1* and the soluble desaturase 1 (*VviSAD-1*)).

**Figure 5:**
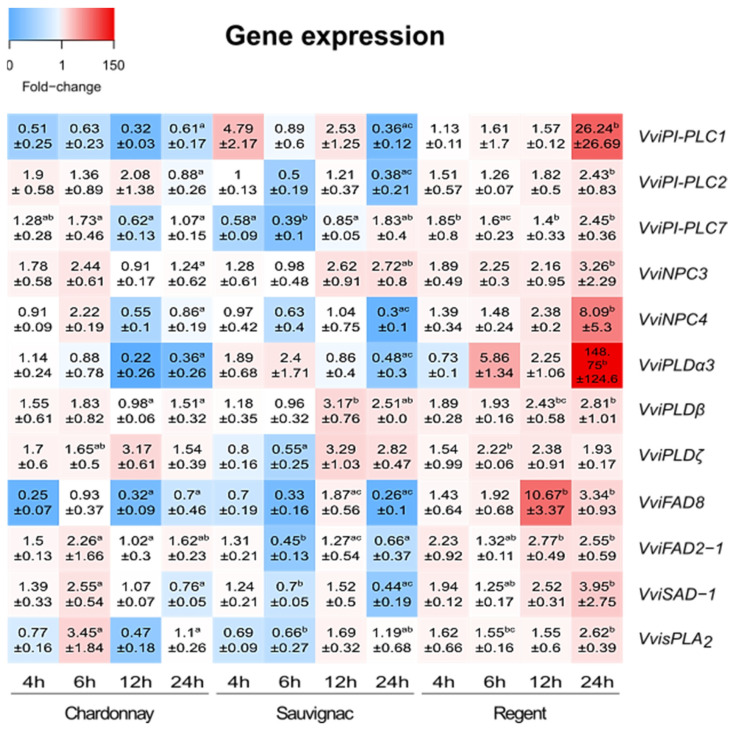
Heatmap representing the expression of *VviPI-PLC1*; *VviPI-PLC2*; *VviPI-PLC7*; *VviNPC3*; *VviNPC4*; *VviPLDα3*; *VviPLDβ*; *VviPLDζ*; *VviFAD8*; *VviFAD2-1*; *VviSAD-1* and *VvisPLA_2_* genes on each of the grapevine cultivars Chardonnay, Regent and Sauvignac. Fold change refers to the comparison between inoculated and mock-inoculated samples at 4, 6, 12 and 24 hpi. Blue indicates lower expression; red indicates higher expression and white indicates no expression (see colour scale). The statistical analysis was performed by Tukey’s multiple comparison tests. Lettering refers to comparisons between the expression of a specific gene between the genotypes at each time point.

The expression profiles of the 12 selected genes (VviPI-PLC1, VviPI-PLC2, VviPI-PLC7, VviNPC3, VviNPC4, VviPLDα3, VviPLDβ, VviPLDζ, VviFAD8, VviFAD2-1, VviSAD-1 and VvisPLA_2_) were analysed by qPCR in *V. vinifera* cv. Chardonnay (susceptible), Regent (tolerant) and Sauvignac (resistant) inoculated with the *P. viticola* isolate NW-10/16 (Figure 5).

In the susceptible genotype, the genes *VviPLDζ* and *VviFAD2-1* were upregulated at all time points, with higher values at 12 and 6 hpi, respectively. *VviPLDα3* decreased its expression over time. *VviPI-PLC1* and *VviFAD8* were downregulated at all time points, except for *VviFAD8* at 6 hpi, which remained at basal levels. All genes were up-regulated at 6 hpi, except for *VviPI-PLC1*, *VviPLDα3* and *VviFAD8*. At 4 hpi, *VviPI-PLC1*, *VviFAD8* and *VvisPLA_2_* were down-regulated, *VviPLDα3* and *VviNPC4* had fold-change near one, whereas the others were upregulated. Lastly, at 24 hpi, *VviPI-PLC7* and *VvisPLA_2_* were at control levels, *VviNPC3*, *VviPLDβ*, *VviPLDζ* and *VviFAD2-1* were upregulated and the rest were downregulated.

Regarding the results observed for the resistant cultivar Sauvignac, *VviPI-PLC2* and *VviNPC4* were near baseline (fold-change at 1), at 4 hpi, *VviPI-PLC7*, *VviPLDζ*, *VviFAD8* and *VvisPLA_2_* were downregulated and the others were upregulated. At 6 hpi, *VviPLDα3* was upregulated and *VviPI-PLC7*, *VviPLDζ*, *VviFAD8* and *VvisPLA_2_* were down-regulated, whereas the remaining half were at control levels. At 12 hpi, two genes were downregulated (*VviPI-PLC7* and *VviPLDα3*), *VviNPC4* had fold-change near one, and the others were all upregulated. Finally, at 24 hpi, *VviPI-PLC7*, *VviNPC3*, *VviPLDβ*, *VviPLDζ* and *VvisPLA_2_* were upregulated, whereas the other seven were downregulated.

All genes were upregulated at all time points in the tolerant cultivar Regent, except for *VviPLDα3*, which was downregulated at 4 hpi, although the higher expression value observed in this analysis was from this same gene at 24 hpi. *VviPI-PLC1*, *VviNPC4* and *VviFAD8* along with *VviPLDα3* presented the most noteworthy differences comparing 4 hpi and 24 hpi, with a significant increase in expression in this last time point.

### 3.3. Fatty Acid Composition of Total Lipids–Gas Chromatography Analysis

**Figure 6:**
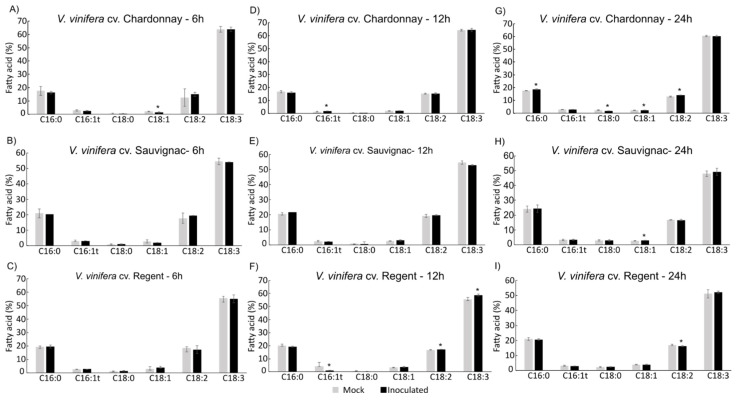
Fatty acid composition of mock-inoculated and inoculated leaves of *V. vinifera* cultivars. Chardonnay, Sauvignac and Regent with *P. viticola* (NW-10/16 isolate), at 6, 12 and 24 hpi. Values correspond to average ± standard deviation, * significantly different *p* < 0.05, *n* = 5.

To investigate differences in the fatty acid profiles of the three *V. vinifera* cultivars (Chardonnay, Sauvignac and Regent) with different degrees of susceptibility and tolerance to *P. viticola*, the FA composition of total leaf lipids was determined at 6 h, 12 h and 24 h post-inoculation, by gas chromatography. The most representative fatty acids were α-linolenic (C18:3), linoleic (C18:2) and palmitic (C16:0) acids (Figure 6).

At the constitutive level, the three cultivars presented distinct FA profiles. The susceptible one, Chardonnay, showed a higher relative amount of C18:3 compared to the more tolerant cultivars Sauvignac and Regent (Figure 6). Upon pathogen challenge, some alterations in the FA profiles were observed, mainly at 12 and 24 h (Figure 6). The most evident changes occurred in Regent at 12 hpi, showing an increase in the C18:2 and C18:3 percentages and a decrease in C16:1t. At 24 hpi, Regent showed a decrease in C18:2 and a tendency for C18:3 accumulation. The susceptible cultivar was mostly affected in the later time point, 24 hpi, demonstrating an increase in C16:0, C18:1 and C18:2 contents and a decrease in C18:0. The cultivar presenting a higher tolerance degree to *P. viticola*, Sauvignac, was the genotype that presented the most constant FA profile along the infection, with no significant changes, with the exception of a slight increase in C18:1 at 24 hpi.

## 4. Discussion

Cellular membranes undergo alterations in their lipid and fatty acid compositions in response to a myriad of biotic and abiotic stresses. Additionally, lipids are indispensable components in plants, providing energy for metabolic processes, acting as structural components of membranes, and playing a central role in plant defence through signalling processes (reviewed in [16]). Lipid signalling events have been highlighted in recent years due to their important role in plant defence against biotic stresses. In response to a pathogen attack, membrane lipids provide substrates for signalling molecules biosynthesis such as free FA, PA, IP3, oxylipins and others [53].

Phospholipases are at the genesis of lipid signalling pathways by their ability to catalyse the hydrolysis of phospholipids into phosphatidic acid (PA), diacylglycerol (DAG), free FA and inositol polyphosphates [54]. These important molecules can act directly as signalling molecules or be channelled to the biosynthetic pathways of other signalling molecules, such as JA [53].

We have previously shown that in the tolerant *V. vinifera* cultivar, Regent, inoculated with a less aggressive *P. viticola* pathovar (not able to overcome the *Rpv3* resistance), a large lipid and FA modulation occurs [12]. These changes concerned mainly chloroplast lipids, whose content increased in infected leaves, as well as an accumulation of C18:3. Once released from membrane lipids by the action of PLA, C18:3 is the substrate of JA, which was also shown to accumulate in response to *P. viticola* [14,15] in the first hours of contact with the pathogen. Polyunsaturated FA contributes to membrane fluidity and their increase upon infection in plastidial lipids could contribute to preventing damage in the photosynthetic machinery [12,13].

In our previous studies, we performed a genome-wide characterisation of the PLA gene family and showed the involvement of some members in grapevine resistance against *P. viticola* [12]. PLDs were also previously characterised [50] but a new version of grapevine genome annotation was released. To identify the main players in grapevine lipid metabolism and signalling pathways, we have analysed both gene expression and fatty acids modulation in response to a highly aggressive *P. viticola* pathovar (NW-10/16). This pathovar was shown to overcome *Rpv3* loci-based resistance (Regent), aggravate disease symptoms in susceptible cultivars (as Chardonnay), and be unable to infect a grapevine genotype that harbours an *Rpv3.1*/*Rpv12* pyramid (Sauvignac) [34]. We have further characterised the PLC and PLD gene families in grapevine.

### 4.1. Grapevine Phospholipases C and D

The grapevine PLC family harbours two groups, the PI-PLC being the larger sub-group with eight members. These results are in accordance with their orthologues in Arabidopsis [41,55], soybean [19] and cotton [56].

PI-PLC acts on phosphatidylinositol (PI), producing inositol trisphosphate (IP_3_) and diacylglycerol (DAG) [57]. In grapevine, these enzymes were predicted to be in the plasma membrane and mitochondria. All the proteins belonging to this sub-family presented four characteristic domains, EF-hand, PI-PLC X, PI-PLC Y and the C2 domain, with the exception of VviPI-PLC4.2 which does not present the EF-hand domain. This domain is a region homologous to the second loop of the Ca^2+^-binding EF-hand of Arabidopsis PLCδ, the so-called “EF-loop” [58] or EF-hand domain [59], that precedes a series of α-helices upstream of the X-domain [58] predicted to participate in catalysis, folding/stability of the enzyme [40] or targeting [60,61]. The conserved X and Y catalytic domains (PI-PLC X and PI-PLC Y) together form a barrel-like structure containing the active site residues [62]. The C2 domain is involved in Ca^2+^-dependent biological processes, unique to plants. This domain is around 130 residues in length and can bind Ca^2+^ and other effectors, including phospholipids, inositol phosphates and proteins, as mentioned in [46,63,64].

The grapevine NPC members were predicted to be in the membranes of several cell compartments, where they can generate DAG from the hydrolysis of both PC and phosphatidylethanolamine (PE), as reviewed in [57]. The grapevine NPC’s proteins showed only one domain, the phosphoesterase domain, required for phospholipase activity [41], and four motifs were predicted: two to be highly conserved, ENRSFD(x)_3_G and TxPNR, and two to be variable, DE(x)_2_G(x)_2_DHV and GxRVP(x)_5_P, as previously described [65,66].

Regarding the *V. vinifera* PLD, the largest subgroup was the C2-PLD, containing 13 proteins, whereas the PXPH-PLD and SP-PLD had only one protein each. These results are in agreement with their PLD orthologues in Arabidopsis [44], soybean [20] and rice [49]. The *V. vinifera* PLD sub-groups presented specific domains at the N-terminal, as predicted in previous works [20,44,49,50]. The members of C2-PLD have a Ca^2+^-dependent phospholipid-binding C2 domain, requiring Ca^2+^ for its enzymatic activity [46]. In contrast to C2-PLD, both *V. vinifera* PXPH-PLD and SP-PLD are Ca^2+^-independent enzymes. The PXPH-PLD showed two specific domains, the Phox homology (PX) and Pleckstrin homology (PH) domains, which are protein regulatory modules involved in cell signalling [44,48]. Finally, the SP-PLD was predicted to have a signal peptide at the N-terminus. All grapevine PLDs possessed two characteristic and structurally conserved HKD motifs: HKD-1, with 8 amino acids (HxK(x)4D), and HKD-2, with 18 amino acids (HxK(x)4D(x)6GSxN), essential for their phospholipase activity [20,44,49,50]. HKD motifs have been demonstrated to have a vital role in catalysis processes [67]. Except for VviPLDφ, the only grapevine SP-PLD, all grapevine PLDs have four other characteristic motifs, three motifs highly conserved in all known PLDs—FIGGIDLCNGRYD, IYIENQ[FY]F and IIGSANINQRS133—and a variable regulatory region for PiP2-binding. The FIGGIDLCNGRYD motif is highly conserved in all PLDs, except in the SP-PLDs, but its specific function has not been described. The IYIENQ[FY]F region also has an important role in catalysis [68]. The IGSANINQRS motif plays an important role in catalysis processes [67]. PiP_2_ has a critical role in PLD activation in mammals and plants, being required for the enzyme activity of some PLDs [68]. The grapevine PLD was predicted to be located mostly in plasma membranes, whereas the VviPLDa was predicted to be located in mitochondria, and the VviPLDφ, the only one containing a signal peptide, was predicted to be secreted. These enzymes can have different substrates. It was shown that C2-PLD can hydrolyse PA from PC, phosphatidylethanolamine (PE) and phosphatidylglycerol (PG), although PX/PH-PLD selectively uses PC as a substrate-producing PA, as reviewed in [43].

### 4.2. Is Lipid Signalling Specifically Linked to Host Genetic Background?

Both lipid and FA composition vary according to the *V. vinifera* genotype, the susceptible genotypes are composed of higher levels of plastidial lipids and PUFAs, and the tolerant/resistant genotypes have higher contents of PA and neutral lipids [12]. Additionally, upon interaction with *P. viticola*, the grapevine genotypes act differently. Only the genotypes presenting a resistant genetic background (*Rpv3.1*) demonstrate lipid and FA modulation as well as an upregulation of the coding genes of enzymes involved in lipid signalling [12,13]. To evaluate if a correlation between the host background and the activation of lipid signalling events is specific, we have analysed the expression profile of 12 genes belonging to the PLC, PLD, PLA and FAD gene families (*VviPI-PLC1*, *VviPI-PLC2*, *VviPI-PLC7*, *VviNPC3*, *VviNPC4*, *VviPLDα3*, *VviPLDβ*, *VviPLDζ*, *VviFAD8*, *VviFAD2-1*, *VviSAD-1* and *VvisPLA_2_*) as well as characterised the modulation of FA during the interaction of the three grapevine genotypes (susceptible, tolerant (*Rpv3.1*) and resistant (*Rpv3.1* and *Rpv12*)) with *P. viticola*.

#### 4.2.1. Lipid Signalling and Accumulation of JA Precursors may Be a Specific Feature of Regent’s Defence

When comparing the global modulation of the three grapevine genotypes, there is a clear difference in the modulation pattern. In Regent, there is an overall increase in gene expression, particularly at 12 and 24 hpi, whereas, in the susceptible and resistant (*Rpv3.1*/*Rpv12*) genotypes, the global modulation pattern is very similar, with several genes being downregulated at 24 hpi.

Considering the PLD genes, the main modulation occurred in Regent, where *VviPLDɑ3* presented a bimodal modulation after *P. viticola* infection, with high expression at 6 h, decreasing by half at 12 h and increasing by 30 times at 24 hpi. The two other PLD genes analysed, *VviPLDβ* and *VviPLDζ* also had their expression increased in all time points, although to lower levels compared to *VviPLDɑ3*. These PLDs could be playing an important role in lipid signalling events by generating PA from the hydrolysis of PC and PE. Phosphatidic acid plays an important role in lipid metabolism, being essential for the novo synthesis of glycerolipids and triacylglycerols [69] and in lipid signalling events, by activating the ABA response, ROS production and JA signalling [70]. The action of PLDs in response to pathogen attacks has been reported. In Arabidopsis, it was demonstrated that PLDɑ1 is involved in ROS generation by producing PA that binds to NADPH oxidase [71]. The ROS accumulation upon pathogen recognition leads to the activation of the plant defence responses [72]. Additionally, PLDβ acts negatively in salicylic acid-mediated response, but positively in JA-meditated response [73]. In plant–fungal interactions, PLDβ leads to a PA increase [73]. In turn, PA can stimulate the biosynthesis of galactolipids by activating monogalactosyldiacylglycerol synthase, which is responsible for the production of JA and oxylipins’ substrates [70]. All the PLCs (*VviPI-PLC1*, *VviPLC2*, *VviPLC7*, *VviNPC3* and *VviNPC4*) analysed had their expression increased mainly at the later time point (24 hpi). In response to pathogens, these grapevine PLCs may be acting in the release of DAG and IP3 from membrane lipids, whereas PI-PLCs act on PI and NPCs act on PC and PE [57]. Inositol 3 phosphate is involved in stomatal closure and oxidative bursts, whereas DAG may be further converted to PA, which has many functions in response to biotic stresses [70]. Moreover, DAG can serve as a precursor of chloroplast membrane lipids (MGDG and DGDG) [41]. The activation of PLC, by the recognition of pathogen-associated molecular patterns, triggers the lipid signalling pathway leading to the activation of the plant defences [74]. In rice, the gene *OsPI-PLC1* is activated in response to the fungus *Magnaporthe grisea*, being reported as essential in the activation of the resistance mechanisms against this pathogen [75]. In tomato, *SlPLC2* and *SlPLC5* had their expression increased in response to fungal elicitors [76] and inoculation with *Cladosporium fulvum* [77] leading to the production of PA as well as the activation of diacylglycerol kinase (DGK) [77]. A high accumulation of MGDG and DGDG in Regent after inoculation with *P. viticola* was previously reported [12]. This event is correlated with the increment of polyunsaturated FAs (PUFAs) and the production of JA, in response to early contact with *P. viticola* [12,13,14]. The increase in C18:2 and C18:3 was also observed in this work, in Regent at 12 hpi. PUFAs can be synthesised in two distinct cell organelles, the endoplasmic reticulum (ER) or the chloroplast. In the ER, C18:2 is synthesised from C18:1 by the action of FAD2, and C18:3 is produced by FAD3. In chloroplasts, these PUFAS are generated by FAD6 and FAD8, respectively, as reviewed in [78]. The increment of the expression of *VviFAD2-1* could be related to an increase in C18:2 production in extraplastidial lipids such as PC. *VviFAD8* showed upregulation at 12 h and 24 hpi, being the higher fold-change identified at 12 hpi. Corroborating this evidence, an increase in C18:3 content is observed in Regent at the same time point. The accumulation of plastidial C18:3 as well as the activation of the FAD8 gene have been reported in the first hours of the interaction of Regent with *P. viticola* [12,13]. The high amount of these PUFAs followed by the activation of *VviPLA* has been related to the JA production that uses C18:3 as a substrate upon its release from membrane lipids by the action of PLAs [18]. The *VvisPLA2* upregulation occurs in Regent, mainly at 24 hpi. Secretory PLAs are involved in the response to pathogens by triggering JA synthesis, stomata opening regulation and H^+^-ATPase activation [79,80]. The upregulation of *VviPLA* suggests that this enzyme may be acting as a defence mechanism response to downy mildew by being involved in JA production and the production of ROS by activating H^+^-ATPase and closing the stomata aperture, which is the *P. viticola* infection starting point.

#### 4.2.2. Both Susceptible and Rpv3.1/Rpv12 Pyramid Genotypes Present Similar Responses

The susceptible cultivar, Chardonnay, presented some similarities to the resistant hybrid, Sauvignac, in the expression analysis results. In general, the majority of the analysed genes did not have their expression increased. In Chardonnay, the gene upregulation occurred mostly at 6 hpi. A slight increase in FA desaturases’ coding genes (*VviFAD2-1* and *VviSAD-1*) may be related to the production of FA conducted by the pathogen demand to uptake nutrients from the host [7]. The secretory PLA, besides having its gene expression increased as well as the PLC and PLD, had a short duration and a low fold-change compared to Regent and Sauvignac. Chardonnay seems not to be able to trigger its lipid signalling process to further activate its defence mechanism, being not able to overcome the infection. In the susceptible cultivar, Chardonnay, the FA profile modifications occur to a higher extent at 24 hpi, revealing an increase in C16:0 content. This FA is synthesised de novo and incorporated in membrane lipids in the chloroplast and the ER. The increase in C16:0 might be related to a higher FA biosynthesis. *Plasmopara viticola* is an obligatory oomycete and keeps the host alive to complete its life cycle. After infection, *P. viticola* is able to switch the grapevine metabolism in order to uptake nutrients from its host [7]. Higher production of C16:0 could be linked to the rearrangement of the host’s metabolism to respond to the high demand for nutrients triggered by the pathogen.

Sauvignac only presents the upregulation of a few genes (*VviPI-PLC1*, *VviNPC3*, *VviPLDβ* and *VviPLDζ*), and for those, the expression is significantly higher than in Chardonnay. This genotype also did not present alterations in the FA profile upon infection, possibly due to its resistance mechanisms, conferred by the presence of two resistance loci, *rp3.1* and *Rpv12*.

## 5. Conclusions

The emergence of new *P. viticola* isolates is changing the grapevine resistance paradigm as these isolates are able to overcome crossing hybrids harbouring resistance loci. The isolate (NW-10/16) is more aggressive and is already able to partially overcome *Rpv3.1* (some sporulation is seen in Regent) resistance but not *Rpv3.1*/*Rpv12* pyramid resistance (no sporulation occurred in Sauvignac). Lipid modulation and signalling were previously shown to be specifically activated in the *Rpv3.1* background when inoculated with a less aggressive pathovar, with no modulation occurring in a susceptible genotype. In the interaction with NW-10/16, despite overcoming the *Rpv3.1*-associated resistance, lipid modulation was also shown to occur. In this case, host lipid modulation and signalling events do not occur as fast and to the extent that they occurred with a less aggressive pathovar, but FAD, lipid signalling-associated phospholipase activation and C18:3 accumulation was shown.

The patterns of fatty acid and lipid modulation and the gene expression of both *Rpv3.1*/*Rpv12* pyramid resistance genotypes and the susceptible genotypes were very similar, suggesting that other mechanisms, rather than lipid modulation and signalling, would be activated. This way, we propose that there could be a link between the genetic background of the host and the activation of a lipid signalling pathway as a defence strategy against *P. viticola*. To further prove this hypothesis, more genotypes with different *Rpv* backgrounds should be analysed.

## Data Availability

Not applicable.

## Supplementary Materials

The following supporting information can be downloaded at: https://www.mdpi.com/article/10.3390/cells12030394/s1; Table S1. Reference and target genes transcripts primer sequences, amplicon length, amplification efficiency, annealing and melting temperature are represented; Table S2. General features of *Vitis vinifera* phospholipases C superfamily. Proposed grapevine PLC nomenclature, gene locus, protein and nucleotide accessions (from NCBI), chromosome location, exon number, protein length, molecular weight (Mw) and isoelectric point (pI) are represented; Table S3. General features of *Vitis vinifera* phospholipases D superfamily. Proposed grapevine PLD nomenclature, gene locus, protein and nucleotide accessions (from NCBI), chromosome location, exon number, protein length, molecular weight (Mw) and isoelectric point (pI) are represented; Table S4. Cis-elements identified in all PLC and PLD grapevine gene family members; Table S5. Subcellular prediction of the *Vitis vinifera* phospholipase C and D proteins; Table S6. Biological process and function of the *Vitis vinifera* phospholipases D’ proteins; Figure S1. Melting curves of targeted genes. A) *EF1α*; B) *UBQ*; C) *VviPI-PLC1*; D) *VviPI-PLC2;* E) *VviPI-PLC7*; F) *VviNPC3*; G) *VviNPC4*; H) *VviPLDα3*; I) *VviPLDβ*; J) *VviPLDζ*; K) *VviFAD8*; L) *VviFAD2-1*; M) *VviSAD-1*; N) *VvisPLA_2_*.
