# Supplementary material for: Grapevine-Associated Lipid Signalling Is Specifically Activated in an Rpv3 Background in Response to an Aggressive P. viticola Pathovar
Source: Cells. 2023 Jan 21;12(3):394. doi: 10.3390/cells12030394 (PMC9913531; doi:10.3390/cells12030394)
Supplement: Supplementary file 1 [file cells-12-00394-s001.zip › FigureS1.pdf]

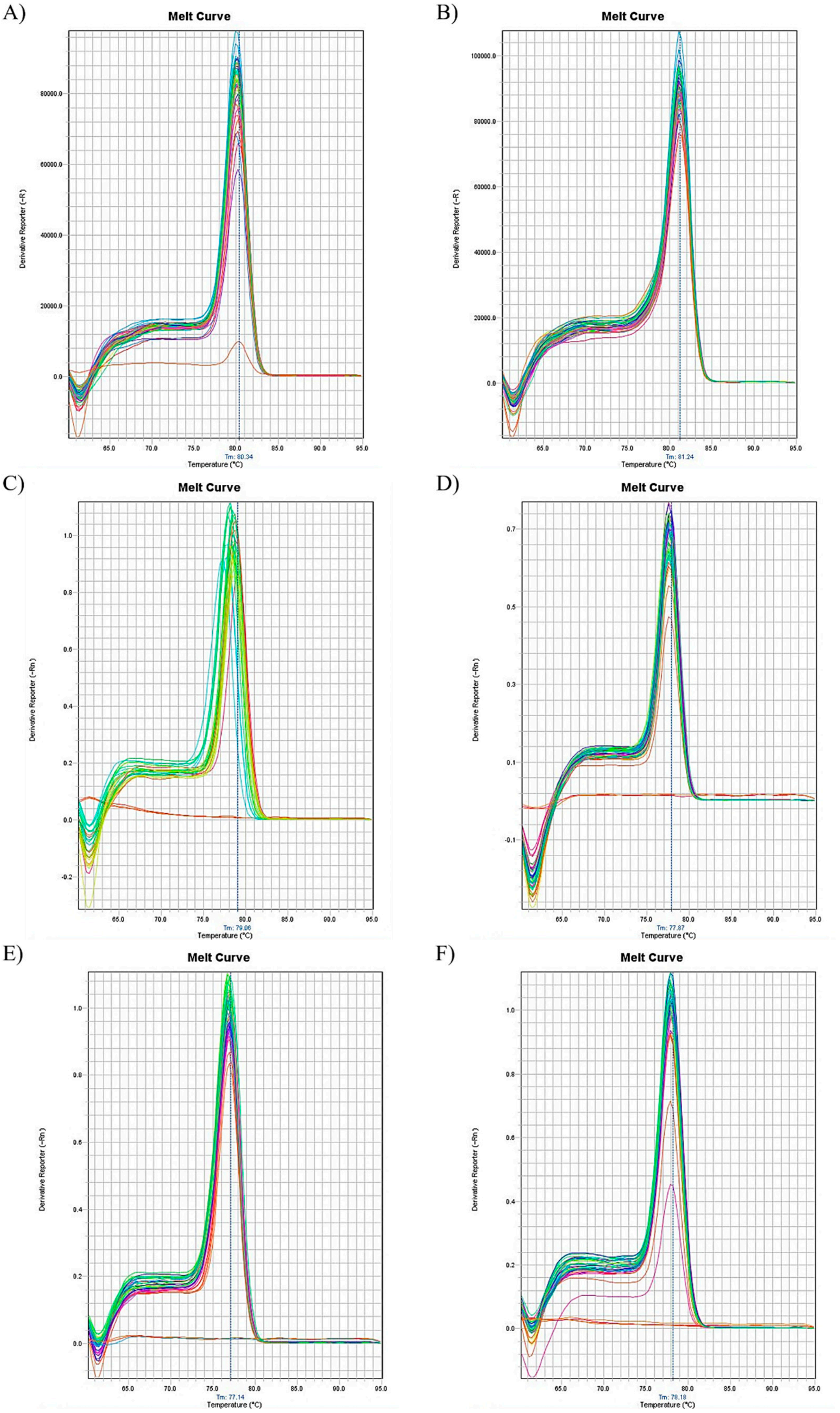
A)
Melt Curve
Derivative Reporter (-R)
Temperature (°C)
Tm: 80.34
B)
Melt Curve
Derivative Reporter (-R)
Temperature (°C)
Tm: 81.24
C)
Melt Curve
Derivative Reporter (-Rn)
Temperature (°C)
Tm: 79.06
D)
Melt Curve
Derivative Reporter (-Rn)
Temperature (°C)
Tm: 77.87
E)
Melt Curve
Derivative Reporter (-Rn)
Temperature (°C)
Tm: 77.14
F)
Melt Curve
Derivative Reporter (-Rn)
Temperature (°C)
Tm: 78.18

G)

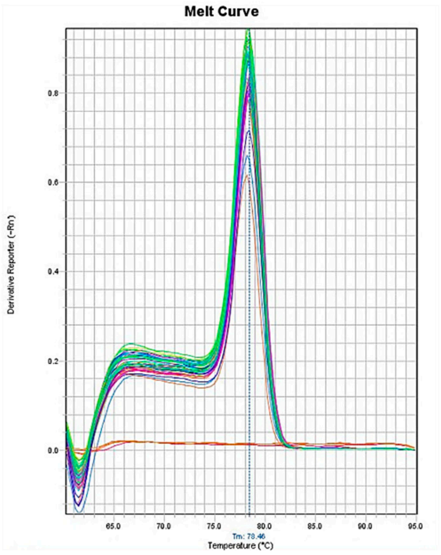
Melt Curve
Derivative Reporter (-Rn)
Tm: 78.46
Temperature (°C)

H)

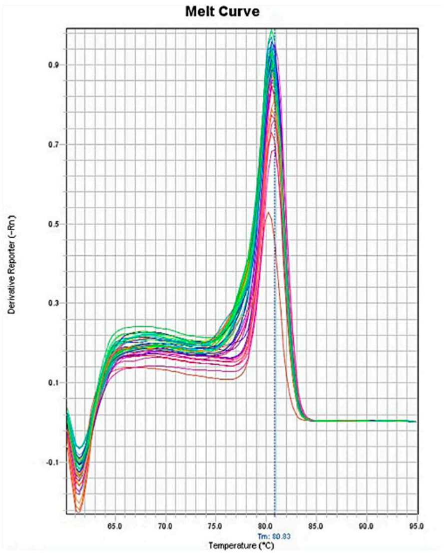
Melt Curve
Derivative Reporter (-Rn)
Tm: 80.83
Temperature (°C)

I)

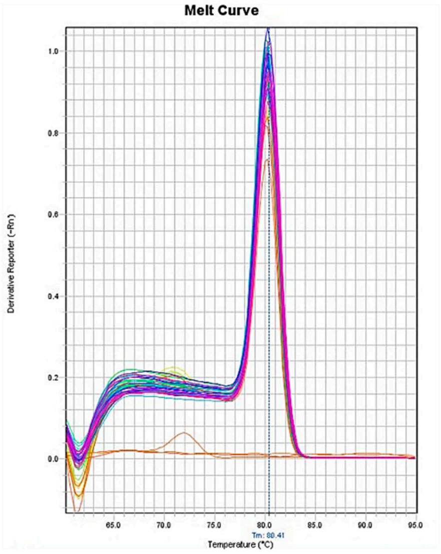
Melt Curve
Derivative Reporter (-Rn)
Tm: 80.41
Temperature (°C)

J)

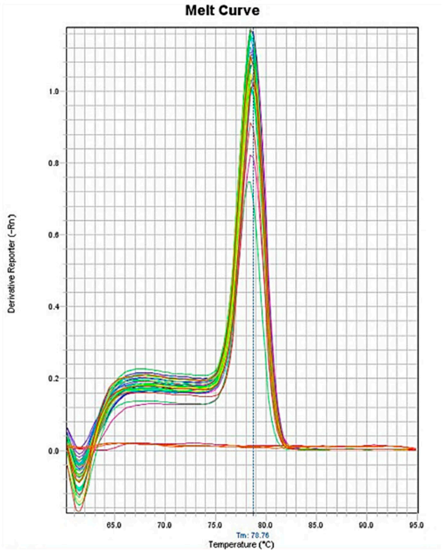
Melt Curve
Derivative Reporter (-Rn)
Tm: 78.76
Temperature (°C)

K)

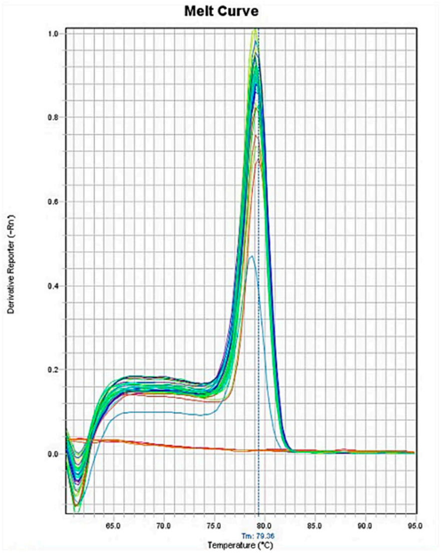
Melt Curve
Derivative Reporter (-Rn)
Tm: 79.36
Temperature (°C)

L)

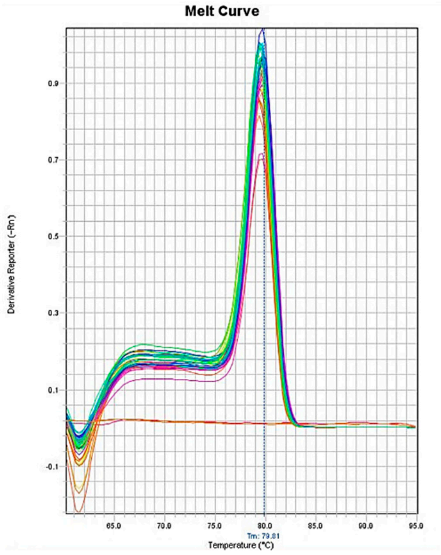
Melt Curve
Derivative Reporter (-Rn)
Tm: 79.81
Temperature (°C)

M) N)

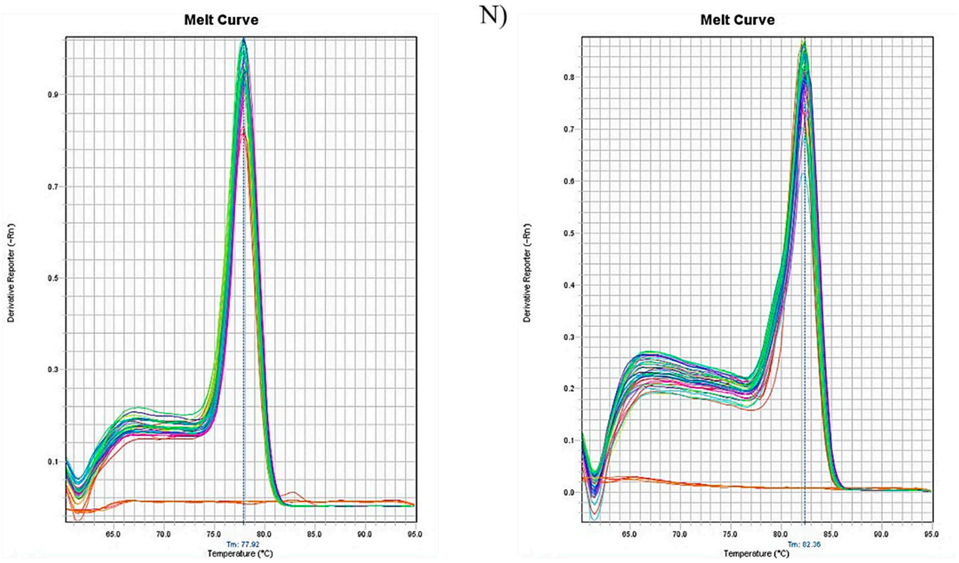

Figure S1 - Melting curves of targeted genes. A) *EF1α*; B) *UBQ*; C) *VviPI-PLC1*; D) *VviPI-PLC2*; E) *VviPI-PLC7*; F) *VviNPC3*; G) *VviNPC4*; H) *VviPLDα3*; I) *VviPLDβ*; J) *VviPLDζ*; K) *VviFAD8*; L) *VviFAD2-1*; M) *VviSAD-1*; N) *VvisPLA*$_2$.
